# Microbial Key Players Involved in P Turnover Differ in Artificial Soil Mixtures Depending on Clay Mineral Composition

**DOI:** 10.1007/s00248-020-01635-1

**Published:** 2020-11-07

**Authors:** Irina Tanuwidjaja, Cordula Vogel, Geertje J. Pronk, Anne Schöler, Susanne Kublik, Gisle Vestergaard, Ingrid Kögel-Knabner, Mirna Mrkonjic Fuka, Michael Schloter, Stefanie Schulz

**Affiliations:** 1 Research Unit Comparative Microbiome Analysis, Helmholtz Zentrum München, Oberschleißheim, Germany; 2 Department of Microbiology, Faculty of Agriculture, University of Zagreb, Zagreb, Croatia; 3 Lehrstuhl für Bodenkunde, Technische Universität München, Freising-Weihenstephan, Germany; 4 Institute of Soil Science and Site Ecology, Dresden University of Technology, Tharandt, Germany; 5 Institute for Advanced Study, Technische Universität München, Garching, Germany; 6 KWR Watercycle Research Institute, Nieuwegein, Netherlands; 7 Department of Health Technology, Technical University of Denmark, Kongens Lyngby, Denmark

**Keywords:** Artificial soils, Bacterial P turnover, Metagenomics, Exopolyphosphatase, Inorganic pyrophosphatase

## Abstract

Nutrient turnover in soils is strongly driven by soil properties, including clay mineral composition. One main nutrient is phosphorus (P), which is known to be easily immobilized in soil. Therefore, the specific surface characteristics of clay minerals might substantially influence P availability in soil and thus the microbial strategies for accessing P pools. We used a metagenomic approach to analyze the microbial potential to access P after 842 days of incubation in artificial soils with a clay mineral composition of either non-expandable illite (IL) or expandable montmorillonite (MT), which differ in their surface characteristics like soil surface area and surface charge. Our data indicate that microorganisms of the two soils developed different strategies to overcome P depletion, resulting in similar total P concentrations. Genes predicted to encode inorganic pyrophosphatase (*ppa*), exopolyphosphatase (*ppx*), and the *pstSCAB* transport system were higher in MT, suggesting effective P uptake and the use of internal poly-P stores. Genes predicted to encode enzymes involved in organic P turnover like alkaline phosphatases (*phoA*, *phoD*) and glycerophosphoryl diester phosphodiesterase were detected in both soils in comparable numbers. In addition, P_o_ concentrations did not differ significantly. Most identified genes were assigned to microbial lineages generally abundant in agricultural fields, but some were assigned to lineages known to include oligotrophic specialists, such as *Bacillaceae* and *Microchaetaceae*.

**Supplementary Information:**

The online version contains supplementary material available at 10.1007/s00248-020-01635-1.

## Introduction

In soils, minerals and microbiota are tightly associated and form highly reactive interfaces [1, 2], which represent activity hotspots. These hotspots can be considered highly structured, heterogeneous, and discontinuous [3]. The resulting soil properties strongly determine water and nutrient availability. The physicochemical and biological characteristics of reactive interfaces are strongly influenced by soil mineral types. For example, swelling clay minerals like montmorillonite are characterized by large specific surface areas, which result in water and nutrient retention. In contrast, non-swelling minerals like illite are characterized by relatively small reactive interfaces [4]. The importance of soil mineral composition as a driver for the formation of interfaces in soils has been proven in experiments with artificial soil mixtures where different combinations of clay minerals (illite and montmorillonite), iron and aluminum oxides (ferrihydrite and boehmite), and charcoal were investigated, while texture and incubation conditions were the same [5–7]. In a 2-year incubation experiment with repeated manure application, Vogel et al. [4] concluded that clay mineral identity was the main driver of microbial community composition. Despite the change in community composition, which was assessed by UPGMA cluster analysis of degenerating gradient gel electrophoresis profiles, the respiration rate, organic matter degradation rate, and quality of organic matter were comparable among the different artificial soil mixtures. Thus, the authors concluded that functionally redundant microbes were present in the different soil mixtures, at least those involved in organic matter degradation.

Phosphorus (P) is an important soil nutrient known to be easily immobilized in soil, and thus sensitive to clay mineral properties [8, 9]. The mineralization and solubilization of P are dependent on hydrolytic enzymes, such as phosphatases, phosphonatases, and C-P lyases [10]. In addition, microorganisms can increase orthophosphate ions in soil solutions or mobility of organic P (P_o_) via direct or indirect mechanisms [8]. Several studies have shown that soil bacteria belonging to different genera including *Pseudomonas*, *Azotobacter*, *Burkholderia*, *Bacillus*, *Rhizobium*, and *Actinomyces* have the ability to increase P availability in soil [11–15]. Clay minerals and their specific surface characteristics have recently been shown to be highly relevant for P binding in soils. At low P concentrations, the structural Al sites of clay minerals are most probably responsible for a comparatively high sorption capacity for P [5]. The specific surface characteristics might directly influence the availability of P in soil and consequently the solubilization and mineralization strategies adapted by microbes [16]. However, the role of clay minerals as drivers for microbiota, which catalyze P transformation processes, is still poorly understood.

In this study, we propose that soils with expandable clay minerals and larger soil surface area can both support a higher diversity of microbiota involved in P turnover and supply more interfaces for P immobilization, thus supporting more P recycling than non-expandable matrices. To test this hypothesis, we used two artificial soils that differ only in clay mineral type and represent a model for an early phase of soil development [4]. One soil contained the expandable clay mineral montmorillonite, the second the non-expandable illite. We used a metagenomics approach to identify microbial P turnover pathways and involved microorganisms.

## Materials and Methods

### Preparation and Maturation of Artificial Soils

Two artificial soil mixtures containing montmorillonite (MT) (Ceratosil WG, Süd-Chemie AG, Moosburg, Germany) or illite (IL) (Inter-ILI Mérnöki Iroda, Hungary) were prepared and incubated. To achieve similar soil texture, each mixture was based on 40-42% sand (Quartz Sand Haltern, H33), 52-54% silt (Millisil W11H, Quarzwerke GmbH, Frechen, Germany), and clay (< 6.3 μm). Montmorillonite (6.3%) and illite (8.0%) were used as clay minerals. Detailed information on the experimental design and model material was previously described [4, 17]. In brief, for each artificial soil mixture, 500 g mixtures were prepared and treated as true replicates throughout. Each mixture was inoculated with a water extractable microbial fraction obtained from the Ap horizon of a Luvisol (Scheyern, Germany) and with a dry and sterilized manure (4.5 wt%, organic carbon (OC) content of 338.5 ± 6.9 mg g^−1^, N content of 30.7 ± 1.6 mg g^−1^, C/N ratio 11.0 ± 0.4) and sieved to < 2 mm as a nutrient source. The artificial soil mixtures were incubated in the dark at 20 °C for 842 days. The water content was kept at 60% of the maximum water holding capacity, which was adjusted at weekly intervals using 0.01 M CaCl_2._. To avoid nutrient depletion, fresh sterile manure was reapplied on day 562 (4.5 wt%, OC content of 165.4 ± 2.7 mg g^−1^, N content of 11.5 ± 0.2 mg g^−1^, and a C/N ratio of 14.4 ± 0.2). Samples were obtained from three independent replicates at the end of the incubation period after 842 days and stored at − 20 °C immediately after sampling.

Analyses of bulk properties and macro-aggregation were performed at the same time point (842 days) and have been recently published [4]. Briefly, macro-aggregates (> 2 mm) differed significantly between the two treatments with higher amounts found in MT (65%) compared to IL (48%). The pH of MT was slightly lower than measured in IL (7.2 ± 0.2 and 7.6 ± 0.3, respectively). The MT had slightly higher OC and N contents than IL soils, but no significant differences were found.

### Measurement of P Concentrations

The bioavailable phosphorus (P_NaHCO3_) was extracted from 4 g dry soil with 40 ml 0.5 M NaHCO_3_. The stable P pools were determined by the ignition-acid extraction method [18]. Briefly, 0.5 g of dry soil was extracted with 25 ml 0.5 M H_2_SO4 before (only stable inorganic P, P_i_) and after ignition at 550 °C (total P, P_total_). In both H_2_SO4 extracts, the molybdate-reactive P was analyzed photometrically (UV-VIS spectral photometer Specord®200, Analytik Jena AG, Jena, Germany) using the molybdenum-blue method. The organic P (P_o_) has been estimated by calculating the difference between P_total_ and P_i_. Each method was carried out in three technical replicates.

### Total Community DNA Extraction, Library Preparation, and Sequencing

Total community DNA was extracted from 0.5 g of each replicate using the NucleoSpin Soil Kit (Macherey-Nagel, Germany) according to the manufacturer’s instructions. To optimize the DNA yield, the proposed protocol with lysis buffer SL2 and enhancer SX was used as described in the user manual. Bead beating was performed twice (5.5 ms^−1^, 30 s) using a Precellys 24 (Bertin Technologies, France). The DNA quality was checked photometrically (Nanodrop ND-1000, Thermo Fisher Scientific, USA) and quantified with the Quant-iT PicoGreen dsDNA Assay Kit (Thermo Fisher Scientific, USA). DNA extracts were stored at − 20 °C.

From each sample, 2 μg of DNA was fragmented using an ultrasonicator (peak instrument power 175 W, duty factor 10%, 200 cycles per burst, treatment time 100 s; E220 Covaris, MA, USA). Fragmented DNA was purified with Agencourt AMPure XP beads (Beckman Coulter, CA, USA) and fragment size was assessed using a Bioanalyzer 2100 (Agilent Technologies, Germany) and Agilent DNA 7500 Kit (Agilent Technologies, Germany). Six metagenomic libraries were prepared from 100 ng DNA per library using the NEBNext Ultra DNA Library Prep Kit (New England BioLabs, MA, USA) according to the manufacturer’s instructions. For adaptor ligation, a 10-fold dilution of NEBNext Adaptor for Illumina with a final concentration of 1.5 μM was used. In the final enrichment, PCR libraries were indexed utilizing NEBNext Multiplex Oligos for Illumina (Dual Index Primers Set 1; New England BioLabs, MA, USA). The PCR reaction contained 2.5 μl of each indexed primer, 25 μl of NEBNext High-Fidelity 2x PCR master mix, and 20 μl of adapter ligated DNA. PCR cycles included an initial denaturation step at 98 °C for 30 s, 6 cycles of denaturation at 98 °C for 10 s, annealing at 65 °C for 30 s, and extension at 72 °C for 30 s followed by a final extension step at 72 °C for 5 min. Metagenomic libraries were electrophoretically size-selected using a Pippin Prep (Sage Science, MA, USA; setting “range mode” to select for 300-580 bp). The final libraries were checked for size using a Bioanalyzer 2100 (Agilent Technologies, Germany) and the Agilent High Sensitivity DNA Kit (Agilent Technologies, Germany) followed by quantification with Quant-iT PicoGreen dsDNA Assay Kit (Thermo Fisher Scientific, USA). Libraries were diluted to 2 nM each. The sample pool was denatured with 0.2 N NaOH, diluted to 10 pM and spiked with 2.5% (v/v) PhiX. Paired-end sequencing was performed on an Illumina MiSeq platform (Illumina, USA), using the MiSeq® 2x 300-cycle V3 kit, following the standard Illumina sequencing protocol. The obtained sequences were deposited to NCBI SRA (Sequence Read Archive) under accession number PRJNA 556907.

### Bioinformatics and Statistical Analysis

Any remaining adaptor sequences or reads shorter than 50 bp and reads with Phred score < 15 were removed from the dataset using AdapterRemoval v2 [19]. Since most improvement in accuracy is achieved by trimming at the level Phred score > 5, a less aggressive trimming approach was chosen to avoid unnecessary read loss [20]. Further processing included removal of PhiX sequences with DeconSeq version 0.4.3 [21]. For taxonomic annotation, high quality fasta sequences were aligned against the NCBI non-redundant (nr) protein sequence data base using DIAMOND [22] and the KEGG database [23] with default settings. Blastn results were mapped and taxonomically and functionally annotated using MEGAN5 (version 5.6.5) [24] with the following parameters: min score = 50, top percent = 10, min support = 1, and min complexity = 0. Sequences of enzymes with corresponding KEGG orthology (KO) numbers involved in phosphorus turnover were extracted from the functionally annotated dataset obtained from the KEGG database and taxonomically annotated by using DIAMOND against NCBI non-redundant (nr) protein sequence database. The 42 enzymes investigated are listed with their corresponding KO numbers in Supplementary Table S1.

Statistical analyses were computed using R [25]. Packages “stats,” “graphics,” “grDevices,” [25] “vegan,” [26] “GUniFrac,” [27] “gridExtra,” [28] “cluster,” [29] “calibrate,” [30] “RColorBrewer,” [31] “gplots,” [32] and “vcd” [33] were used. The metagenomic datasets were subsampled to the lowest number of reads per sample by using the rarify function from the “GUniFrac” package. Significant differences for the taxonomic and functional annotation were determined by unpaired *t* test, where *p* values were adjusted by Bonferroni test.

## Results

### P Concentrations of the Two Artificial Soils

The results of the P_total_, P_o_, P_i_, and P_NaHCO3_ measurements are summarized in Table 1. P_total_ did not significantly differ between the two soils. P_i_ was significantly higher in MT (304 mg kg^−1^) than IL (264 mg kg^−1^), while P_o_ concentrations did not significantly differ between the two soil mixtures, and were only slightly higher in IL (120 mg kg^−1^). P_NaHCO3_ concentrations were significantly lower in IL (134 mg kg^−1^) than MT (153 mg kg^−1^).

**Table 1:** Phosphorus concentrations of artificial soils containing MT and IL after 842 days of incubation. The first manure addition took place when the experiment started and the second after 562 days of incubation. The values are shown as mean with standard deviation of three independent replicates (*n* = 3). Data include total P (P_total_), stable organic P (P_o_), stable inorganic P (P_i_), and bioavailable P (P_NaHCO3_). Asterisks indicate significant differences as revealed by *t* test (*p* < 0.05, *n* = 3)

	MT	IL	*p* value	1st manure	2nd manure
P_total_ (mg/kg)	396 ± 20	384 ± 29	0.229	10,516 ± 157	2755 ± 110
P_NaHCO3_ (mg/kg)	153 ± 8	134 ± 9	0.003*	870 ± 27	387 ± 25
P_i_ (mg/kg)	304 ± 35	264 ± 14	0.007*	8546 ± 145	1976 ± 35
P_o_ (mg/kg)	93 ± 43	120 ± 28	0.130	1970 ± 296	778 ± 131

### Phylogenetic Annotation of Artificial Soil Metagenomes

**Fig. 1:**
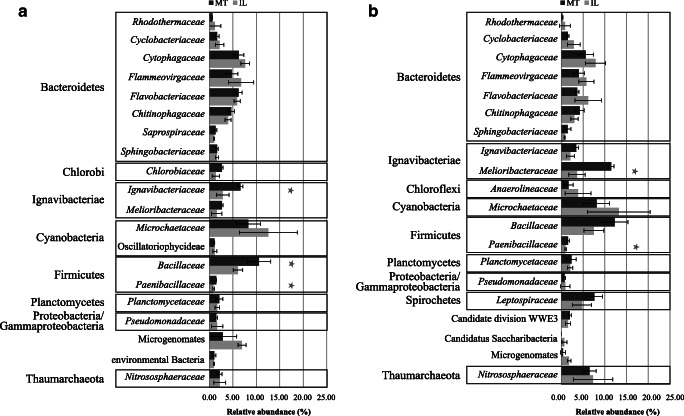
Relative abundance of overall prokaryotic families (**a**) and families potentially involved in P turnover (**b**) in the metagenomes of two artificial soils. Sequences were aligned against the NCBI-nr database and annotated with MEGAN5. Relative abundances were calculated based on the number of all assigned reads. Twenty most abundant taxa are shown. Asterisks indicate significant differences (*p* < 0.05, *n* = 3)

To identify microbial key players involved in P turnover, a metagenomic approach was used. All metagenomic datasets were randomly subsampled to the lowest number of reads per sample (*n* = 3,074,662). The coverage of metagenomes based on the read redundancy values as calculated by the Nonpareil algorithm ranged between 0.657 and 0.773 (data not shown). Of all reads, 55.84% could be annotated using the NCBI non-redundant protein sequence database. Later relative abundances were calculated based on assigned reads. Of those, 93.72% were bacterial reads, 4.21% eukaryotic, and 2.07% archaeal. Overall, the obtained reads were assigned to 477 prokaryotic families. Only 7 of those predicted families showed a relative abundance > 1.0% in both artificial soils, namely *Microchaetaceae* (4.50%), *Bacillaceae* (3.51%), *Cytophagaceae* (2.95%), *Flavobacteriaceae* (2.57%), *Flammeovirgaceae* (2.48%), *Ignavibacteriaceae* (1.96%), and *Chitinophagaceae* (1.81%) (Fig. 1a). Besides those 7 families, an additional 339 families were shared between both artificial soils, but in lower abundance. To detect significant differences between the prokaryotic communities in MT and IL, the abundance of all families was compared. To be more stringent, only taxa with a relative abundance of at least 0.005% in one of the samples were included. The relative number of significantly different reads at the family level is shown in the Supplementary Table S2. In general, 54 bacterial families differed significantly in the number of assigned reads. Among the 20 most abundant families, *Ignavibacteriaeceae*, *Bacillaceae*, and *Paenibacillaceae* were significantly higher in MT (Bonferroni adjusted unpaired *t* test, *p* = 0.010, *p* = 0.045, and *p* = 0.003, respectively) while no bacterial family was significantly more abundant in IL.

### Functional Annotation of Artificial Soil Metagenomes

The functional annotation of reads was performed by aligning the subsampled dataset against the KEGG database [23] and visualizing the results using MEGAN5 [24]. In total, 92,430 reads were assigned to genes predicted to code for enzymes involved in P turnover. Rarefaction analysis of KO numbers associated with P turnover revealed comparable functional richness for both metagenomes of MT and IL (Supplementary Figure S1).

**Fig. 2:**
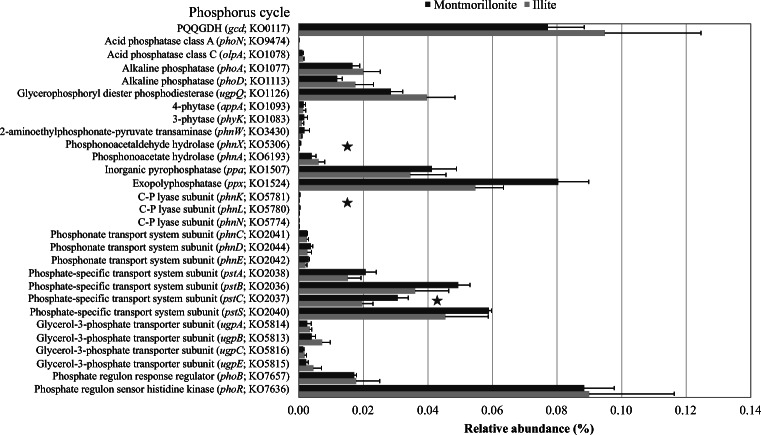
Relative abundance of prokaryotic genes predicted to be involved in phosphorus turnover in both artificial soils. Obtained sequences were aligned against KEGG database and annotated with MEGAN5. Relative abundances were calculated based on the number of all assigned KEGG reads. Asterisks indicate significant differences (*p* < 0.05, *n* = 3) in the number of annotated reads between the investigated soils

Of the 42 investigated genes, 29 could be detected in both soils (Fig. 2, Supplementary Table S1). The majority of reads could be assigned to the genes involved in phosphorus transport and uptake (33,720), followed by mineralization (26,461), regulatory systems (18,774), and solubilization (13,525). Among those predicted genes, three were significantly more abundant in MT: *phnX*, *phnK*, and *pstC*. In addition, other predicted genes were more abundant in MT, but not on a significant level of 5%. Those genes included further subunits of the phosphate-specific transport system (*pstSCAB*), inorganic pyrophosphatase (*ppa*), and exopolyphosphatase (*ppx*, *p* = 0.059). Genes coding for the glycerol-3-phosphatase transport system (*ugpBAEC*), alkaline phosphatases (*phoA*, *phoD*), glycerophosphoryl diester phosphodiesterase (*ugpQ*), and quinoprotein glucose dehydrogenase (*gcd*) were more abundant in IL, although this was not significant. Genes associated with P regulation (*phoR*, *phoB*) showed nearly identical relative abundances in both soils.

### The Microbiota Driving the Phosphorus Turnover in MT and IL Soils

**Fig. 3:**
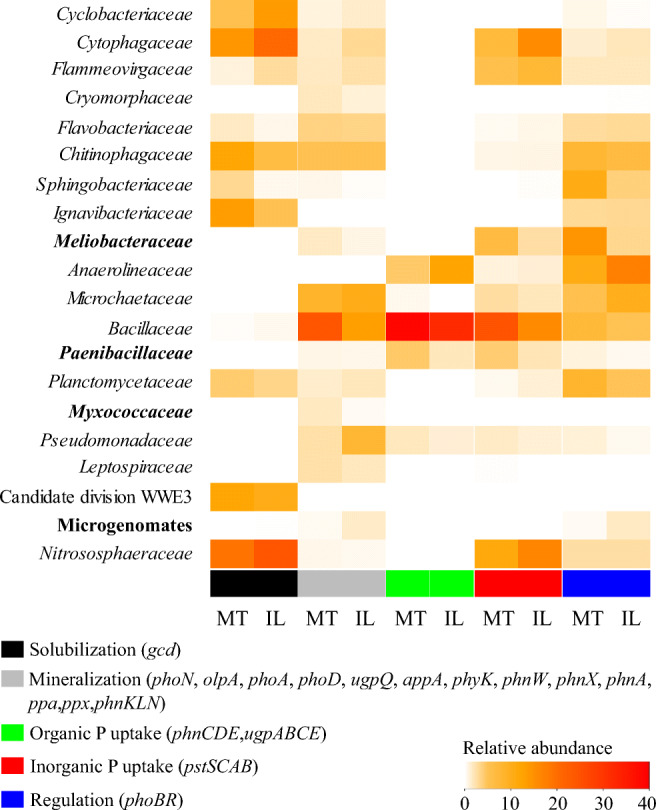
Relative abundance of families involved in P turnover in the montmorillonite (MT) and illite (IL) artificial soils. Obtained sequences were assigned on the functional level by aligning against the KEGG database and on taxonomic level against the NCBI-nr database. Relative abundances were calculated based on the number of reads assigned to P turnover. Twenty most abundant families are depicted. Shown are mean values of all genes involved in solubilization, mineralization, organic uptake, inorganic uptake, and regulation. Families marked bold represent families that differ significantly between artificial soils (*p* < 0.05, *n* = 3)

To predict key drivers of phosphorus turnover, the 29 genes detected in both soils were extracted from the KEGG annotated dataset, and taxonomically annotated using DIAMOND and NCBI non-redundant (nr) protein sequence database. In general, families predicted to be involved in most P turnover processes in both soils mostly belonged to the highly abundant families, such as *Nitrososphaeraceae*, *Cytophagaceae*, *Bacillaceae*, *Chitinophagaceae*, and *Flammeovirgaceae* (Fig. 1b, Fig. 3, Supplementary Figure S2). More specifically, it was predicted that P solubilization in MT was driven by *Ignavibacteriaceae*, *Cytophagaceae*, *Chitinophagaceae*, candidate division WWE3, and *Nitrososphaeraceae*. The mineralization of P was predicted to be dominated by *Bacillaceae* and *Microchaetaceae* and uptake by *Anaerolinaceae*, *Bacillaceae*, and *Meliobacteriaceae* in MT. Similar families were potentially involved in P turnover in IL.

**Fig. 4:**
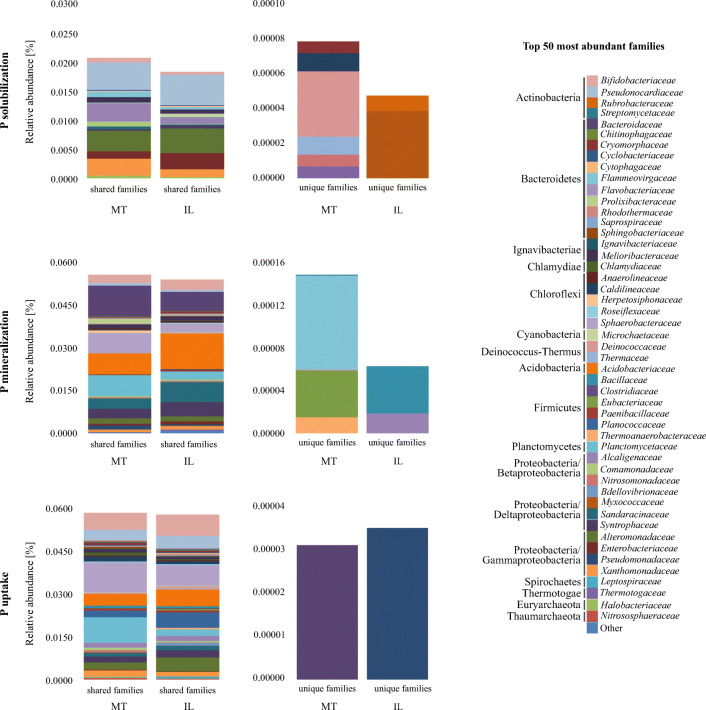
Distribution of shared and unique families involved in P turnover in the montmorillonite (MT) and illite (IL) artificial soils. Obtained sequences were functionally assigned using the KEGG database and taxonomically using the NCBI-nr database. Shown are mean values of the relative abundance of the fifty most abundant families involved in P solubilization, mineralization, and uptake. Relative abundances were calculated based on the number of all assigned reads

Interestingly, most of the families predicted to be involved in P turnover were shared between the two soils (Fig. 4). Among the 50 most abundant families, 17 were similar in IL and MT for solubilization, 41 for mineralization, and 43 for the uptake of P. The relative abundance of unique families was generally very low. In IL, unique families potentially involved in P solubilization were assigned to *Prolixybacteraceae* and *Halobacteriaceae*, and for P mineralization to *Ignavibacteriaceae* and *Sphaerobacteraceae*. Unique families in MT predicted to be involved in P solubilization (*Streptomycetaceae*, *Saprospiraceae*, *Deinococcaceae*, *Thermaceae*, *Paenibacillaceae*, and *Pseudomonadaceae*) and P mineralization (*Chlamydiaceae*, *Herpetosiphonaceae*, *Thermaceae*, *Syntrophaceae*, and *Thermotogaceae*) were slightly more diverse when compared to IL. Regarding P uptake, only one family per soil was predicted to be unique, namely *Leptospiraceae* in MT and *Cryomorphaceae* in IL. Although both soils had many families in common, the relative abundance of 13 bacterial families that were predicted to be involved in P turnover was significantly different, with only *Meliobacteriaceae* (*p* = 0.002) and *Paenibacillaceae* (*p* = 0.042) belonging to the 20 most abundant families (Supplementary Table S3). Only *Bacillaceae* potentially covered all processes in both soils, while the other families were predicted to drive specific transformation steps in P turnover (Fig. 3).

## Discussion

### Microbial Diversity and Community Establishment Are Affected by Soil Mineral Composition

The effects of mineral composition on the establishment of microbial communities in the initial phase of soil development have been described in a number of publications focusing on several short-term [34, 35] and long-term [4] experiments. For example, Ding et al. [35] showed for the same artificial soil mixtures we used that genera belonging to the phyla Proteobacteria, Firmicutes, Actinobacteria, and Bacteroidetes were significantly higher in IL compared to MT after 90 days of incubation. We demonstrated in our study based on metagenomic sequences that the relative abundances of 35 families were significantly higher in MT, while only 19 were significantly higher in IL. This is in line with our assumption that MT containing soil mixtures promote a more diverse community. Vogel et al. [4] showed that aggregation in matured artificial soils differed depending on clay minerals, while total nutrient concentrations were similar. P_total_ concentrations in our experiment also confirmed their findings (Table 1). After 842 days, the content of macro-aggregates was higher in MT than IL, suggesting a faster aggregate turnover in artificial soils with IL. Moreover, differences in the microbiome composition might be explained by the protective influence of mineral surfaces on organic matter and consequently lower mineralization rates inducing the slower release of nutrients in MT, shown by the higher OC content as well as significantly higher P_o_ and bioavailable P_NaHCO3_ concentrations in MT. We assume that this might have influenced members of *Bacillaceae*, *Ignavibacteriaceae*, and *Chlorobiaceae* that have the ability to utilize recalcitrant compounds from organic matter [36, 37]. In contrast, a weaker immobilization potential in IL could promote a faster mineralization of OC by members of Bacteroidetes like *Cytophagaceae*, Flammeovirgaceae, and *Pseudomonadaceae*, which are frequently found in copiotrophic environments [38] and may have benefited from the addition of manure, which introduced a huge amount of nutrients (Table 1). Moreover, Vogel et al. [4] also observed the increase of *Pseudomonas* species after the second manure addition.

### Microbial P Turnover in Montmorillonite and Illite Containing Artificial Soils

Although the last nutrient addition was more than 300 days ago, both soils showed surprisingly high concentrations of available inorganic P (Table 1), comparable for example to forest soils [39]. This might be linked to the low microbial respiration rates at the time of sampling [4] indicating low microbial activity and highly efficient strategies for the remobilization of P from the microbial biomass, as the community composition significantly changed in the 300 days after the last manure addition [4]. Furthermore, the preliminary limitation of other nutrients like nitrogen and carbon might have caused high remaining P concentrations, which is underlined by very low C:P and N:P ratios of less than 3.8 and 34.6, respectively (calculation based on [4] and Table 1). These values are much lower than those observed from Cleveland and Liptzin [40], who postulated a well constrained C:P ratio of 186:1 and N:P ratio of 13:1. This is further in line with findings of Heuck et al. [41], who demonstrated that under C limitation, organic P resources are mineralized with the aim to take up the released C first, which explains higher C:P ratios in our experiment. Moreover, the use of internally stored P might be an alternative, for example in acidocalcisomes. In both soils, genes predicted to encode the phosphate-specific transport system (pst) were highly abundant, while no reads were assigned to the phosphate inorganic transporter (pitA). This suggests that P is a highly competitive nutrient in those soils, as this transporter is typically expressed under P starvation [42]. It is not surprising that bacterial families predicted to be involved in P cycling belonged to the most abundant families, as P mobilization and uptake are of high importance in nutrient-limited soils. Members of *Bacillaceae* were among the most abundant bacteria in both soils and have the potential to perform all processes of P turnover. These results nicely confirm previous data from cultured members of *Bacillaceae*. For example, *Bacillus* spp. include well-known phosphorus-solubilizing bacteria (PSB). They harbor a variety of P-solubilizing mechanisms (e.g., changing pH, excretion of organic acids, siderophores, and phosphatase) [15]. Another advantage is the ability of *Bacillaceae* to sporulate under unfavorable conditions, thus helping them to survive and outcompete other microbiota in MT and IL soils [36].

Since P is one of the most important macronutrients, essential in energy metabolism and other cellular processes, enzymes related to the use of external P sources are harbored by a wide range of microorganisms [43], which we also show in this study (Fig. 1b). Genes predicted to be involved in external P turnover were assigned to microbiota belonging to Bacteroidetes, Firmicutes, Proteobacteria, and Planctomycetes in both soils, which is comparable to findings of Grafe et al. [44] in mature agricultural soils. This is not surprising, as the artificial soils had been inoculated with a microbial community extracted from an agricultural soil at the beginning of the maturation phase. In contrast, Bergkemper et al. [45] found that most reads assigned to P turnover in metagenomes from P-depleted forest soils belonged to the Acidobacteria (Acidobacteriales, Solibacterales), Actinobacteria (Actinomycetales), and Proteobacteria (Rhodospirillales, Burkholderiales). This difference could be due to the complexity of forest soil and the resulting differences in physicochemical properties, as well as to the different mineral composition, which selects for different families. In the MT, the most abundant predicted genes associated with P mineralization were ppa and ppx; the respective enzymes are predominantly involved in the utilization of inorganic P. The ppa gene codes for an inorganic pyrophosphatase, an enzyme that hydrolyzes polyphosphate compounds (poly-P), and ppx for an exopolyphosphatase which in turn releases inorganic P from poly-P chains [46] in prokaryotes under the condition of P starvation [47]. Thus, a first mechanism in overcoming P starvation might be the use of internal P storage pools like acidocalcisomes, which might have been developed after the addition of manure. Acidocalcisomes are well known and widespread both in prokaryotes and eukaryotes [48]. Moreover, MT has a higher storage capacity than IL due to its bigger soil surface area. Thus, inorganic P from external sources like manure might persist longer. Interestingly, in MT, the reads predicted to code for the *ppx* gene (exopolyphosphatase) were exclusively assigned to members of Cyanobacteria. Recent studies have shown that the expression of the *ppx* gene allows Cyanobacteria to adapt to the environmental fluctuation of P and C [47]. Additionally, it has been demonstrated that exopolysaccharides can be used to solubilize P. As Cyanobacteria are well known as exopolysaccharide producers, it might be an advantage in MT soils where more reaction sites are available [49]. An additional advantage of some Cyanobacteria could be their potential capability to fix nitrogen and CO_2_ [50].

In contrast, the relative abundance of predicted genes involved in P mineralization did not significantly differ in the two soils. This was further accompanied by comparable P_o_ concentrations. This included genes coding for alkaline phosphatases (ALP), namely *phoA* and *phoD*, and *ugpQ*, which coded for a glycerophosphoryl diester phosphodiesterase. While PhoA includes mainly phosphomonoesterase, PhoD shows phosphodiesterase activity as well [51]. UgpQ has phosphodiesterase activity and hydrolyzes glycerophosphoryl diesters to glycerol-3-phosphate (G3P). In line with that, we also detected reads, which were assigned to all four subunits of the glycerol-3-phosphate transporter system (*ugpBAEC*), whose relative abundance exceeded those detected in other studies [44, 45]. This could allow the utilization of organic P sources in nutrient-depleted systems. The detection of predicted ALP genes was higher than that of the predicted acid phosphatase genes in both soils. However, this was not surprising as both soils had a pH of 7.7 at the beginning of the experiment [17] and of 7.2 (MT) and 7.6 (IL) after 842 days of incubation [4], which is still above the optimal pH for acid phosphatase activities [52]. Moreover, Fraser et al. [53] demonstrated that the addition of manure might increase the abundance of the phoD community as well as the ALP activity. Genes coding for the Pho regulon [54, 55] that controls *phoA* and *phoD* gene expression were also highly abundant in both soils, which was in line with the high abundance of *pstS*, underlining again the need for a strict control of P utilization.

Interestingly, the predicted quinoprotein glucose dehydrogenase (PQQGDH), potentially involved in P solubilization, was among the top three abundant genes in both soils. Its relative abundance was even higher compared to a study from agricultural soils [44], which regularly obtained organic fertilizers. Solubilization processes so far have been mostly described for P-rich systems [45]. However, the P_NaHCO3_ concentration and the total P_i_ concentration (Table 1) suggest that there is still a high potential to solubilize P from the stable inorganic fraction.

## Conclusion

The predicted key players involved in P transformation in our study were among the dominant taxa, which underlines the importance of P acquisition in both artificial soil mixtures. The dominant families predicted to be involved in P turnover like *Bacillaceae* and *Microchaetaceae* are perfectly adapted to harsh environments with the ability to rest as spores or acquire additional nutrients like C and N by fixation. Our data indicate that organic P is an important source in both artificial soil mixtures, as many reads were assigned to genes potentially involved in the effective use of organic P sources, including different alkaline phosphatases and glycerophosphoryl diester phosphodiesterase. These might be involved in the mineralization of still available P_o_ introduced with the manure, or P_o_ released from the microbial biomass, whose composition significantly changed in more than 300 days after the last manure addition. In MT, which has a larger soil surface area, the relative abundance of the predicted *pstC* gene was significantly increased compared to IL. This gene belongs to the high affinity phosphate-specific transporter (*pstSCAB*) system acting under P starvation. Moreover, the potential to use internal P sources, by the breakdown of poly-P species, was greater in MT compared to IL.

However, the data represent the system’s potential, which was predicted on the basis of metagenomic assignments for certain processes and do not confirm the actual turnover. In order to deepen the insight into P turnover mechanisms and to identify active microbial communities involved in the processes, RNA and proteome-based studies and additional experiments should be employed.

## Supplementary Information

ESM 1 (PDF 829 kb)

## Data Availability

The metagenomic sequencing data have been uploaded to the sequencing read archive (SRA) under the bioproject number PRJNA556907. All other data generated or analyzed during this study have been included in this published article (and its supplementary information files).
