# Supplementary material for: Microbial Key Players Involved in P Turnover Differ in Artificial Soil Mixtures Depending on Clay Mineral Composition
Source: Microb Ecol. 2020 Nov 7;81(4):897–907. doi: 10.1007/s00248-020-01635-1 (PMC8062357; doi:10.1007/s00248-020-01635-1)
Supplement: Supplementary file 1 — (PDF 829 kb) [file 248_2020_1635_MOESM1_ESM.pdf]

Microbial keyplayers involved in P turnover differ in artificial soil mixtures depending on clay mineral composition

Irina Tanuwidjaja[1,4] , Cordula Vogel[2,5], Geertje J. Pronk[2,3,6], Anne Schöler[1], Susanne Kublik[1], Gisle A. Vestergaard[1,7], Ingrid Kögel-Knabner[2,3], Mirna Mrkonjic Fuka[4], Michael Schloter[1,2], Stefanie Schulz[1*]

Affiliation

[1] Research Unit Comparative Microbiome Analysis, Helmholtz Zentrum München, Oberschleißheim, Germany

[2] Lehrstuhl für Bodenkunde, Technische Universität München, Freising-Weihenstephan, Germany

[3] Institute for Advanced Study, Technische Universität München, Garching, Germany

[4] Department of Microbiology, Faculty of Agriculture, University of Zagreb, Zagreb, Croatia

[5] Institute of Soil Science and Site Ecology, Dresden University of Technology, Tharandt, Germany

[6] Ecohydrology Research Group, University of Waterloo, Waterloo, Ontario, Canada

[7]Department of Biology, University of Copenhagen, Universitetsparken 15, DK-2100 Copenhagen, Denmark

*Corresponding author:

Dr Stefanie Schulz

Email: stefanie.schulz@helmholtz-muenchen.de

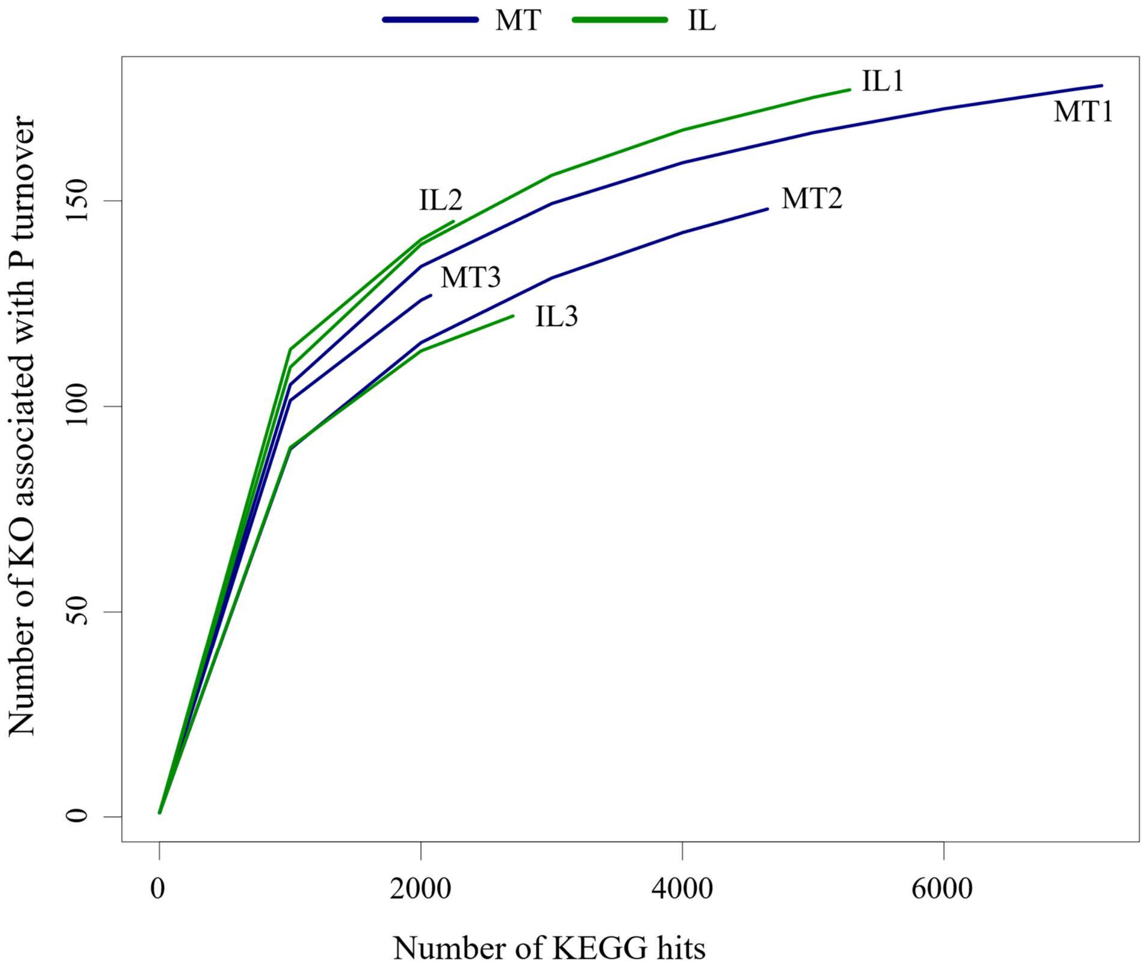

Supplementary Figure S1: Rarefaction curves based on KO numbers associated with P turnover in montmorillonite and illite. Depicted is the number of KEGG hits associated with P turnover against all KEGG hits.

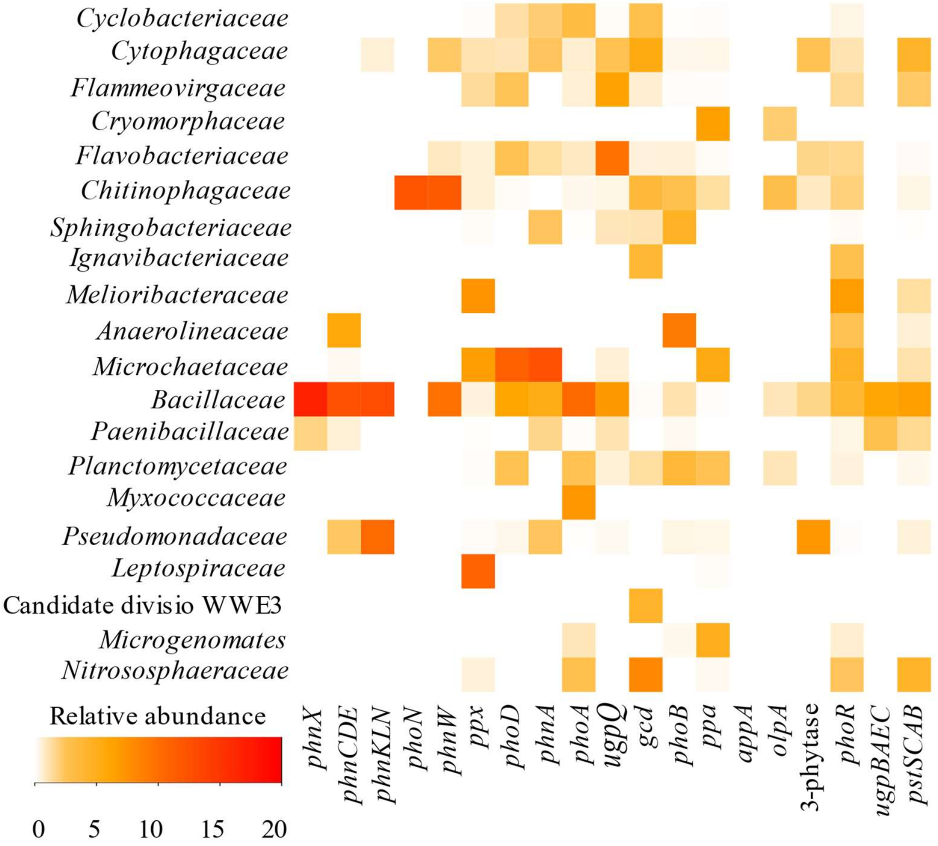

Supplementary Figure S2: The distribution of microbial genes associated with P turnover across twenty most abundant families. Obtained sequences were assigned on the functional level by aligning against KEGG database and on taxonomic level against NCBI-nr database. Twenty most abundant families are depicted. The values represent mean relative abundance of both soils of assigned sequences per gene.

Supplementary Table S1: Genes and corresponding KEGG orthology numbers (KO) investigated and detected in this study.

**Investigated enzymes**	***Corresponding gene***	**KEGG KO number**	**Detected in dataset**	**Reference**
PQQGDH	*gcd*	KO0117	+	[1]
Acid phosphatase class A	*phoN*	KO9474	+	[2]
Acid Phosphatase class B	*aphA*	KO3788	-	[2]
Acid Phosphatase class C	*olpA*	KO1078	+	[2]
Alkaline phosphatase	*phoA*	KO1077	+	[3]
Alkaline phosphatase	*phoD*	KO1113	+	[4]
Alkaline Phosphatase	*phoX*	no KEGG KO number available	-	[5]
Glycerophosphoryl diester phosphodiesterase	*ugpQ*	KO1126	+	[6]
Phosphotriesterase	*opd*	KO7048	-	[7]
4-phytase	*appA*	KO1093	+	[8]
3-phytase	*phyK*	KO1083	+	[9]
2-aminoethylphosphonate-pyruvate transaminase	*phnW*	KO3430	+	[10]
Phosphonoacetaldehyde hydrolase	*phnX*	KO5306	+	[10]
Phosphonoacetate hydrolase	*phnA*	KO6193	+	[10]
Inorganic pyrophosphatase	*ppa*	KO1507	+	[11]
Exopolyphosphatase	*ppx*	KO1524	+	[12]
C-P lyase subunit	*phnF*	KO2043	-	[10]
C-P lyase subunit	*phnG*	KO6166	-	[10]
C-P lyase subunit	*phnH*	KO6165	-	[10]
C-P lyase subunit	*phnI*	KO6164	-	[10]
C-P lyase subunit	*phnJ*	KO6163	-	[10]
C-P lyase subunit	*phnK*	KO5781	+	[10]
C-P lyase subunit	*phnL*	KO5780	+	[10]
C-P lyase subunit	*phnM*	KO6162	-	[10]
C-P lyase subunit	*phnN*	KO5774	+	[10]
C-P lyase subunit	*phnO*	KO9994	-	[10]
C-P lyase subunit	*phnP*	KO6167	-	[10]

Phosphate inorganic transporter	*pit*	KO3306	-	[13]
Phosphonate transport system subunit	*phnC*	KO2041	+	[14]
Phosphonate transport system subunit	*phnD*	KO2044	+	[14]
Phosphonate transport system subunit	*phnE*	KO2042	+	[14]
Phosphate-specific transport system subunit	*pstA*	KO2038	+	[14]
Phosphate-specific transport system subunit	*pstB*	KO2036	+	[14]
Phosphate-specific transport system subunit	*pstC*	KO2037	+	[14]
Phosphate-specific transport system subunit	*pstS*	KO2040	+	[14]
Glycerol-3-phosphate transporter subunit	*ugpA*	KO5814	+	[14]
Glycerol-3-phosphate transporter subunit	*ugpB*	KO5813	+	[14]
Glycerol-3-phosphate transporter subunit	*ugpC*	KO5816	+	[14]
Glycerol-3-phosphate transporter subunit	*ugpE*	KO5815	+	[14]
Phosphate regulon response regulator	*phoB*	KO7657	+	[14]
Phosphate regulon sensor histidine kinase	*phoR*	KO7636	+	[14]
PhoR/PhoB Inhibitor Protein (PhoU)	*phoU*	KO2039	-	[14]

Supplementary Table S2: Relative abundance of significantly different sequences annotated to the prokaryotic families in artificial soils are shown. Relative abundances were calculated based on the number of all assigned reads. Values are shown as mean with standard deviation of three independent replicates (n = 3). 4 archaeal families were detected and are marked in grey. Asterisks indicate families whose abundance exceeds 0.005%. Sequences were aligned against NCBI Non-redundant protein (NCBI-nr) database and annotated with MEGAN5.

**Family**	**MT**	**IL**	**Top 20 taxa**	**p-value**
Actinomycetaceae	0.013 ± 0.003	0.023 ± 0.005		0.039
Dermacoccaceae	0.002 ± 0.001	0.003 ± 0.000		0.038
Porphyromonadaceae	0.431 ± 0.038	0.344 ± 0.034		0.042
Prevotellaceae	0.221 ± 0.018	0.163 ± 0.024		0.027
Amoebophilaceae	0.226 ± 0.038	0.047 ± 0.026		0.003
environmental Bacteroidetes	0.079 ± 0.008	0.033 ± 0.010		0.003
Cryomorphaceae	0.242 ± 0.037	0.410 ± 0.039		0.006
Ignavibacteriaceae	6.601 ± 0.526	2.837 ± 1.332	*	0.010
Caldisericaceae	0.012 ± 0.003	0.004 ± 0.001		0.017
Criblamydiaceae	0.024 ± 0.002	0.012 ± 0.005		0.018
environmental Acidobacteriales	0.003 ± 0.000	0.012 ± 0.004		0.023
unclassified Acidobacteria	0.210 ± 0.008	0.117 ± 0.042		0.019
Chitinivibrionaceae	0.025 ± 0.006	0.012 ± 0.005		0.048
Bacillaceae	10.576 ± 2.505	6.093 ± 1.017	*	0.045
Paenibacillaceae	1.465 ± 0.017	0.874 ± 0.161	*	0.003
Peptoniphilaceae	0.094 ± 0.012	0.045 ± 0.022		0.027
Symbiobacteriaceae	0.015 ± 0.001	0.010 ± 0.002		0.024
Halanaerobiaceae	0.024 ± 0.000	0.017 ± 0.002		0.002
Thermoanaerobacterales Family IV. Incertae Sedis	0.015 ± 0.002	0.008 ± 0.002		0.023
Fusobacteriaceae	0.112 ± 0.014	0.073 ± 0.020		0.049

Gemmatimonadaceae	0.201 ± 0.046	0.076 ± 0.031	0.018
unclassified Rhizobiales	0.016 ± 0.002	0.010 ± 0.002	0.028
Anaplasmataceae	0.022 ± 0.003	0.012 ± 0.004	0.023
Candidatus Midichloriaceae	0.001 ± 0.001	0.003 ± 0.001	0.039
Gallionellaceae	0.007 ± 0.000	0.013 ± 0.003	0.021
unclassified Methylophilales	0.003 ± 0.000	0.001 ± 0.001	0.010
environmental Desulfobacterales	0.013 ± 0.002	0.007 ± 0.002	0.033
Desulfomicrobiaceae	0.004 ± 0.001	0.007 ± 0.001	0.036
Desulfurellaceae	0.054 ± 0.004	0.026 ± 0.014	0.033
environmental delta subdivision	0.015 ± 0.001	0.008 ± 0.003	0.014
Myxococcaceae	0.198 ± 0.005	0.122 ± 0.035	0.020
environmental Myxobacteria	0.001 ± 0.000	0.000 ± 0.000	0.025
Labilitrichaceae	0.125 ± 0.020	0.075 ± 0.019	0.035
unclassified Myxococcales	0.052 ± 0.002	0.033 ± 0.006	0.006
Syntrophobacteraceae	0.189 ± 0.003	0.083 ± 0.046	0.016
Syntrophorhabdaceae	0.046 ± 0.005	0.028 ± 0.003	0.006
Acidithiobacillaceae	0.007 ± 0.002	0.013 ± 0.003	0.035
Pseudoalteromonadaceae	0.125 ± 0.003	0.106 ± 0.006	0.008
Psychromonadaceae	0.009 ± 0.001	0.016 ± 0.002	0.007
Chromatiaceae	0.110 ± 0.007	0.162 ± 0.024	0.023
Ectothiorhodospiraceae	0.110 ± 0.016	0.182 ± 0.011	0.003
Hahellaceae	0.061 ± 0.009	0.094 ± 0.011	0.017
Pasteurellaceae	0.043 ± 0.003	0.033 ± 0.002	0.015
Gallaecimonas	0.004 ± 0.000	0.009 ± 0.003	0.033
Spongiibacter	0.023 ± 0.003	0.012 ± 0.002	0.006
Thiobacillus prosperus	0.019 ± 0.001	0.009 ± 0.003	0.003
unclassified Gammaproteobacteria	0.056 ± 0.013	0.096 ± 0.011	0.015
unclassified Vibrionales	0.005 ± 0.001	0.002 ± 0.001	0.044
unclassified Zetaproteobacteria	0.013 ± 0.002	0.006 ± 0.001	0.004
Entomoplasmataceae	0.003 ± 0.001	0.001 ± 0.000	0.037

Caldithrix	0.500 ± 0.039	0.243 ± 0.093	0.011
candidate division CPR2	0.008 ± 0.007	0.022 ± 0.001	0.030
candidate division Zixibacteria	0.732 ± 0.060	0.344 ± 0.203	0.034
Latescibacteria	0.007 ± 0.001	0.003 ± 0.001	0.010
Pyrodictiaceae	0.001 ± 0.000	0.002 ± 0.000	0.007
Fervidicoccaceae	0.009 ± 0.002	0.002 ± 0.001	0.005
Methanococcaceae	0.093 ± 0.009	0.042 ± 0.025	0.031
Methanospirillaceae	0.003 ± 0.000	0.015 ± 0.006	0.032

Supplementary Table S3 Relative abundance of significantly different sequences annotated to the prokaryotic families involved in P turnover in artificial soils are shown. Relative abundances were calculated based on the number of all assigned reads. Values are shown as mean with standard deviation of three independent replicates (n = 3). 1 archaeal family was detected and marked in grey. Asterisks indicate families whose abundance exceeds 0.005%. Sequences were aligned against NCBI-nr database and annotated with MEGAN5.

**Family**	**MT**	**IL**	**Top 20 taxa**	**p-value**
Melioribacteraceae	11.492 ± 0.635	3.604 ± 1.869	*	0.002
Paenibacillaceae	1.530 ± 0.287	0.981 ± 0.147	*	0.042
Eubacteriaceae	0.627 ± 0.146	0.094 ± 0.106		0.007
Peptococcaceae	0.132 ± 0.042	0.285 ± 0.055		0.019
Thermoanaerobacteraceae	0.796 ± 0.123	0.342 ± 0.138		0.013
Gemmatimonadaceae	0.111 ± 0.042	0.013 ± 0.022		0.022
Rhodobiaceae	0.005 ± 0.008	0.216 ± 0.093		0.017
Myxococcaceae	1.181 ± 0.193	0.194 ± 0.089		0.001
Alteromonadales genera incertae sedis	0.105 ± 0.008	0.159 ± 0.016		0.007
Ectothiorhodospiraceae	0.030 ± 0.036	0.117 ± 0.032		0.034
Oceanospirillaceae	0.000 ± 0.000	0.067 ± 0.026		0.012
Thiotrichaceae	0.264 ± 0.066	0.084 ± 0.045		0.017
unclassified Microgenomates	0.412 ± 0.572	1.794 ± 0.418		0.028
Caldisphaeraceae	0.077 ± 0.114	0.325 ± 0.092		0.043

References

1. Cleton-Jansen AM, Goosen N, Fayet O, Van de Putte P (1990) Cloning, mapping, and sequencing of the gene encoding Escherichia coli quinoprotein glucose dehydrogenase. J Bacteriol 172:6308–6315. https://doi.org/10.1128/jb.172.11.6308-6315.1990
2. Rossolini GM, Schippa S, Riccio ML, et al (1998) Bacterial nonspecific acid phosphohydrolases: Physiology, evolution and use as tools in microbial biotechnology. Cell Mol Life Sci 54:833–850. https://doi.org/10.1007/s000180050212
3. Torriani A (1960) Influence of inorganic phosphate in the formation of phosphatases by Escherichia coli. Biochim Biophys Acta 38:460–469
4. Eder S, Shi L, Jensen K, et al (1996) A Bacillus subtilis secreted phosphodiesterase/alkaline phosphatase is the product of a Pho regulon gene, phoD. Microbiology 142:2041–2047. https://doi.org/10.1099/13500872-142-8-2041
5. Monds RD, Newell PD, Schwartzman JA, O'Toole GA (2006) Conservation of the Pho regulon in Pseudomonas fluorescens Pf0-1. Appl Environ Microbiol 72:1910–1924. https://doi.org/10.1128/AEM.72.3.1910
6. Brzoska P, Boos W (1988) Characteristics of a ugp-encoded and phoB-dependent glycerophosphoryl diester phosphodiesterase which is physically dependent on the ugp transport system of Escherichia coli. J Bacteriol 170:4125–4135. https://doi.org/10.1128/jb.170.9.4125-4135.1988
7. McDaniel CS, Harper LL, Wild JR (1988) Cloning and sequencing of a plasmid-borne gene (opd) encoding a phosphotriesterase. J Bacteriol 170:2306–2311. https://doi.org/10.1128/jb.170.5.2306-2311.1988
8. Golovan S, Wang G, Zhang J, Forsberg CW (2000) Characterization and overproduction of the Escherichia coli appA encoded bifunctional enzyme that exhibits both phytase and acid phosphatase activities. Can J Microbiol 46:59–71

9. Sajidan A, Farouk A, Greiner R, et al (2004) Molecular and physiological characterisation of a 3-phytase from soil bacterium Klebsiella sp. ASR1. Appl Microbiol Biotechnol 65:110–118. https://doi.org/10.1007/s00253-003-1530-1

10. McGrath JW, Chin JP, Quinn JP (2013) Organophosphonates revealed: New insights into the microbial metabolism of ancient molecules. Nat Rev Microbiol 11:412–419. https://doi.org/10.1038/nrmicro3011

11. Lahti R, Pitkäranta T, Valve E, et al (1988) Cloning and characterization of the gene encoding inorganic pyrophosphatase of Escherichia coli K-12. J Bacteriol 170:5901–5907. https://doi.org/10.1128/jb.170.12.5901-5907.1988

12. Akiyama M, Crooke E, Kornberg A (1993) An exopolyphosphatase of Escherichia coli: The enzyme and its ppx gene in a polyphosphate operon. J Biol Chem 268:633–639

15. Elvin CM, Dixon NE, Rosenberg H (1986) Molecular cloning of the phosphate (inorganic) transport (pit) gene of Escherichia coli K12. Mol Gen Genet 204:477–484. https://doi.org/10.1007/bf00331028

16. Hsieh YJ, Wanner BL (2010) Global regulation by the seven-component Pi signaling system. Curr Opin Microbiol 13:198–203. https://doi.org/10.1016/j.mib.2010.01.014
